# Rapid Decoupled Electrochemical Reduction of CO_2_ to Syngas

**DOI:** 10.1002/cssc.202502728

**Published:** 2026-07-11

**Authors:** Mark Potter, Hamza Annath, Craig G. Armstrong, Marina Goupalova, Enrico Andreoli, Kathryn E. Toghill

**Affiliations:** ^1^ Department of Chemistry Lancaster University Lancaster UK; ^2^ Department of Chemical Engineering Swansea University Swansea UK

**Keywords:** electrocatalysis, electrochemistry, redox chemistry, sustainable chemistry

## Abstract

Decoupled electrochemical reactions provide a scalable strategy for electrosynthesis, especially as the demand for renewably sourced chemicals intensifies. Here, we report a rapid, decoupled electrochemical reduction of CO_2_ to syngas (CO and H_2_), using a Cr(II) PDTA complex with a highly reducing potential (in aqueous media, −0.7 V vs. RHE) in conjunction with a gold–poly(vinyl alcohol)/carbon nanocomposite catalyst. We demonstrate the efficient discharge of the CO_2_‐saturated Cr(II) solution, generating syngas at a rate of 30–60 mA mg^−1^ of catalyst, achieving comparable conversion rates to state‐of‐the‐art gas diffusion electrode (GDE) assemblies reported in the literature at ‘mild’ CO_2_ reduction potentials of ca. −0.8 V versus RHE. Systematic optimisation of the catalyst composite enabled control over CO/H_2_ ratios, highlighting a promising route for the scalable, green production of Fischer–Tropsch feedstocks via redox mediated CO_2_ electroreduction.

## Graphical abstract

Using a gold‐carbon catalyst and chelated‐chromium redox mediator, electrochemically decoupled reduction of CO_2_ to CO was achieved. A range of H_2_‐CO ratios were obtained by altering the amount of poly vinyl alcohol capping agent added during catalyst synthesis. Prolonged experiments indicated a comparable mass activity to conventional electrocatalysis at a relatively mild reducing potential, highlighting the viability of this radically different approach.

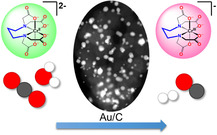

## 1 Introduction

The use of fossil fuel‐derived hydrocarbons as a source of both primary energy and industrial chemicals has led to ever‐increasing anthropogenic carbon dioxide emissions, which continues to have a driving effect on both global temperature and weather severity [1]. It is imperative that alternative technologies be implemented as soon as possible to limit the environmental cost. Electrochemical reactions driven by renewably generated electricity propose one possible solution to this issue. Electrochemical carbon dioxide reduction (ECO_2_R) offers both long‐term energy storage and a carbon neutral source of many important industrial chemicals. The production of many simple hydrocarbons ranging from C_1__–_C_3_ has been demonstrated utilising a range of catalyst materials, however the most active and selective catalysts tend to favour the two‐electron reduction products CO or HCOOH. Widespread adoption of ECO_2_R remains limited by a variety of issues including low energy efficiency, poor catalyst stability and complicated electrolyser configurations [2, 3, 4].

As an emerging approach to electrochemical reactions, redox mediators can be employed to decouple the electrochemical reaction from the electrode surface by acting as charge transport vectors. This was first reported for water splitting by Amstutz et al. in 2014, where a vanadium‐cerium redox flow battery was modified to discharge over Mo_2_C and IrO_2_ catalysts for decoupled hydrogen and oxygen evolution respectively [5]. Our recent article reported the first successful decoupled electrochemical carbon dioxide reduction (DECO_2_R), wherein a chromium complex with a particularly negative redox potential in aqueous solution was used alongside a tailored bismuth nanomaterial to enable the selective reduction of CO_2_ to formate [6]. While this initial proof of concept demonstrated that the process works in principle, it was limited by poor overall charge efficiency and low turnover owing to the low mediator concentration. Furthermore, as a product, formate requires the additional complication of needing to be extracted from the mediator solution, which adds complexity and expense to the process. Targeting instead gaseous products, such as CO, would allow for spontaneous product separation which would be much more practical to implement. As a product, CO offers to be much more relevant to the ongoing objective of carbon neutrality. Presently, CO is sourced from the processing of fossil fuels, however renewably sourced CO could be used alongside renewably generated H_2_ in the production of a wide range of hydrocarbons using the Fischer–Tropsch process [7, 8].

Of the CO producing catalysts, the noble metals gold and silver tend to be the most selective and active [9, 10]. Gold in particular sits atop the volcano plot for CO selectivity due to favourable binding with the key reduction intermediate *COOH, while having poor binding with the formate‐favouring *OCHO intermediate and the competing HER *H intermediate [11, 12]. Gold nanoparticles, often supported on carbon substrates, are commonly reported to reduce CO_2_ to CO with high selectivity and efficiency at relatively low overpotentials [13, 14, 15, 16]. As such, gold based catalysts are the focus of this work.

Decoupled approaches to synthesis offer the potential advantage in the rapid conversion of CO_2_ to CO_2_R to products, by optimising reaction conditions towards more favourable kinetics. However; with the use of a liquid phase redox mediator, it is imperative that the reaction products be readily separated from the reaction medium to make the process practicable. With this in mind, we sought to extend the CO_2_ reduction of the preceding work, enabled using a Cr complex with a highly reducing redox potential of −0.7 V versus RHE which is close to the limit of water stability on carbon felt, and high stability in carbonate solution, to achieve only gaseous products. The use of this relatively mild potential requires the use of catalysts with low overpotentials. From the literature we identified a gold/ polyvinyl alcohol (PVA) catalyst reported to have such low onset potentials (<−0.8 V vs. RHE) for syngas production by Ma et al. [13] Combining our reduced mediator with the carbon supported gold nanoparticles with PVA capping resulted in the rapid evolution of syngas (CO and H_2_), the composition of which could be varied using different amounts of PVA in nanoparticle synthesis. Furthermore, an initial assessment into scaling up the process was performed to determine how it might compare to state‐of‐the‐art conventional catalysis such as GDE flow cells [17].

## 2 Results and Discussion

### 2.1 Catalyst Choice

Promising catalyst material for CO_2_ conversion to CO was selected from existing literature, with a focus on high selectivity at low overpotentials. It was decided that supported nanoparticles would be a better choice as catalysts, as it will be easier to separate them from the mediator solution post reaction. While it was envisioned that the unique kinetics of the decoupled system would not require the catalyst/support to be electronically conductive, the vast majority of materials reported for CO_2_R use conductive supports/additives like carbon black. One such gold‐based material, which also utilised PVA as a capping agent, was reported to have CO selectivity above 90% for the full potential range −0.4 to −0.8 V versus RHE [13]. Synthesis proved facile, and when deployed as a catalyst for DECO_2_R resulted in a CO selectivity over 40%. This material was chosen as the basis for further optimisation of the DECO_2_R process towards gas products.

Polymer binders are required to ensure the catalyst remains attached to the electrode in conventional electrocatalysis, however they often provide beneficial effects beyond improving the mechanical stability of the catalyst layer. Nafion remains the most popular choice of binder, due to its high durability and ability to conduct protons. However, recent studies have suggested that Nafion can have detrimental effects on the CO_2_ reduction reaction. In some examples of copper‐catalysed CO_2_R, using too much Nafion binder reduced selectivity towards the preferred C_2_ product ethylene, in turn increasing the proportion of H_2_, and to a lesser extent CO, produced [18].

Despite the ubiquity of Nafion, many alternatives have been explored [19]. The chemical environment provided by the binders can typically be classified into two main categories based on their functionalisation; hydrophobic and hydrophilic. The way this functionality interacts with the catalyst surface can influence product selectivity by either stabilising or destabilising the competing reaction intermediates. For the case of the gold catalysed reduction of CO_2_ to CO, it was found that the CF_2_ groups in the hydrophobic binders PTFE and PVDF improved CO selectivity primarily by destabilising the adsorbed hydrogen intermediate, while the hydrophilic binders PVA and PAA improved CO selectivity by also stabilising the adsorbed CO_2_R intermediate [20]. At the lower end of the overpotential range between −0.5 to −0.7 V versus RHE all the binders, except PAA, resulted in similar performance within error. Only at the higher overpotentials up to −1.0 V versus RHE was a categorical improvement observed, in which the hydrophobic binders were notably more selective towards CO than the hydrophilic ones.

As it is presently employed however, DECO_2_R does not require the use of binders for mechanical stability. That however does not mean that the beneficial effects of these polymers on the chemical environment cannot be exploited. Having successfully achieved CO as a major product using PVA to improve selectivity, a deeper exploration on the influence of the PVA loading on selectivity was performed. It was previously reported that increasing the amount of PVA coverage on the catalyst further improved CO selectivity during H‐cell bulk electrolysis experiments performed at a relatively mild potential of −0.4 V versus RHE [13]. A series of PVA loadings between 0 and 30 wt% were prepared by increasing the amount of PVA present during nanoparticle formation. Here the % PVA quoted is defined as the wt% of PVA added to the solution compared to the total weight of gold and carbon.

**FIGURE 1:**
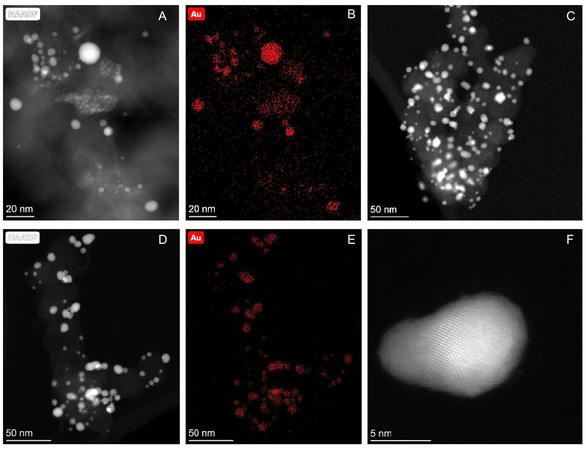
STEM images of Au/C 20 wt% prepared by rapid reduction of HAuCl_4_ by NaBH_4_ in the absence of PVA (A) and the presence of 15 wt% PVA (D), followed by impregnation onto carbon black. Respective gold EDX (B), (E). Au/C 20 wt% with 15 wt% displaying high dispersity and narrow particle size range (C). Single gold nanoparticle displaying crystal lattice (F).

High‐resolution STEM imaging of the PVA free material was compared to the 15% wt% PVA material, which indicated that the presence of PVA during nanoparticle formation resulted in a narrower range of particle sizes (Figure 1). Further, the dispersity of the gold over the carbon is much higher when PVA was present. For the PVA free sample, large patches of carbon free of gold nanoparticles were observed, while for the 15% wt% PVA sample, very little carbon free of gold was observed. This can be rationalised by the observation that gold nanoparticle impregnation occurred much more rapidly in the sample free of PVA than in samples that contained PVA. The rapid impregnation likely resulted in poor dispersity because the hydrophobic carbon could not be mixed in fast enough, while nanoparticles capped with PVA were captured more slowly, allowing for the carbon to be thoroughly mixed during impregnation.

### 2.2 Conventional Electrochemical Performance

To test the material against what has previously been reported [13], it was prepared for conventional electrode voltammetry and bulk electrolysis. The PVA loading of the material used in these tests was 15 wt%. Linear sweep voltammetry, (Figure S11) with 1 M KCl supporting electrolyte displays a clear diffusion limited peak in the presence of CO_2_, suggesting good activity and selectivity for CO_2_R. Reduction appeared to onset around −0.3 V versus RHE, peaking at −0.7 V versus RHE. The peak was less defined in KHCO_3_ electrolyte, however a clear shift in onset potential and increase in current plateau was observed when switching from N_2_ to CO_2_ atmosphere (Figure S12). LSVs performed in electrolyte containing 10 mM Cr(III) PDTA (1,3‐propylenediamine‐N,N,N′,N′‐tetraacetate) displayed no clear current response as a result of its presence, however the overlap in potential is a good indication that DECO_2_R using this material is energetically favourable.

Bulk electrolysis in a conventional H‐cell was performed using the Au/C 15 wt% PVA across a range of applied potentials close to that which the charged Cr(II) mediator could be expected to provide electrons. Results, summarised in Table S1, indicated a reasonably high selectivity for CO, although not as high as previously reported [13]. The highest selectivity was achieved at −0.656 V versus RHE, with CO making up 90% of the products. The observed current density was, however, much lower than the previously reported 98.6 mA cm^−2^ at −0.77 V versus RHE, at only 2.4 mA cm^−2^ at −0.756 V versus RHE, though the literature value is surprisingly high for an H‐cell study. Indeed, the ECSA normalised response given by Ma et al. for the high surface area electrode was ca. 0.15 mA cm^−2^ for CO conversion at −0.4 V versus RHE compared to the measured ca. 4 mA cm^−2^ [13]. Hydrogen was the only observed side product of the reaction. The use of Nafion binder proved necessary for stability of the catalyst layer and could not be omitted; the presence of which may affect catalyst performance relative to the decoupled system described hereafter.

### 2.3 Decoupled CO_2_R Performance

**FIGURE 2:**
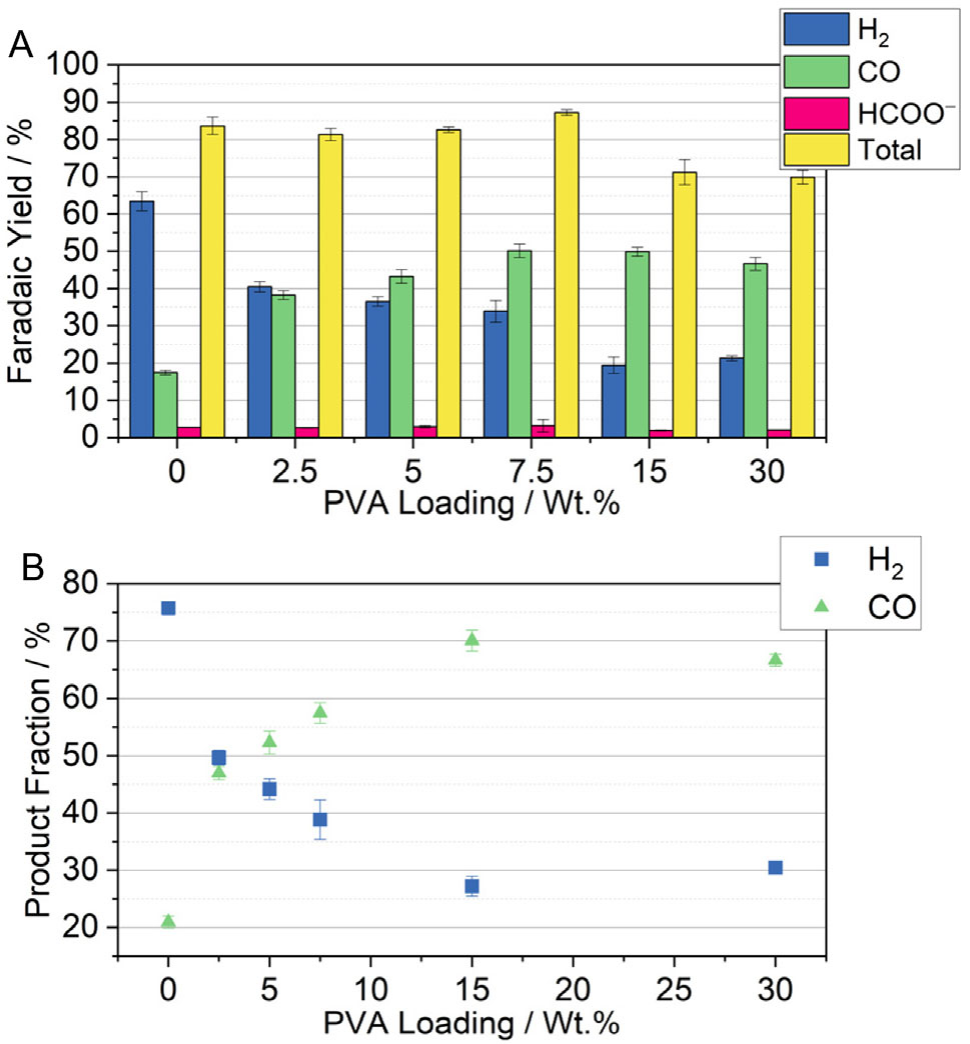
(A) The average FY of H_2_ (blue), CO (green), HCOO(H) (pink), and in total (yellow) measured when 20 mL aliquots of 8.8 mM Cr PDTA were discharged over the Au/C PVA heterogenous catalysts, with error bars indicating the standard deviation within each triplicate set. (B) Product fraction of the observed products provided by H_2_ and CO across the range of PVA loadings.

A series of catalysts was deployed for DECO_2_R by a batch method in which an aliquot of charged Cr(II)PDTA mediator (prepared as described in the SI) was added to a Schlenk flask containing the desired catalyst under a CO_2_ atmosphere. Figure 2A summarises the results showing the absolute percentage Faradaic yields (electrons required to produce measured products /electrons provided by mediator addition, FY) of the three reduction products with respect to the PVA loading of the gold catalyst. Figure 2B concerns the selectivity of the total products towards CO or H_2_, referring to a product fraction with respect to PVA loading. Starting with Au/C catalyst prepared in the absence of PVA, a CO selectivity of just 21% (16% absolute yield) was observed. The addition of even a small amount of PVA during nanoparticle synthesis, just 2.5 wt%, increased the CO selectivity up to 47% (38% absolute yield), effectively producing a syngas of about 1:1 H_2_:CO. Doubling this to 5.0 wt% pushed CO selectivity to 52%, and this trend continued linearly up until 15 wt% PVA, at which point the CO selectivity was 70%, producing a syngas of around 1:2 H_2_:CO. Further increasing the PVA content to 30%, whereby extrapolation of the observed trend would predict 100% CO, actually saw a slight decline in CO selectivity, falling back to 67%. FY remained in the low to mid 80% range for the lower wt% samples, while it began to fall at 15 wt% to just over 70%, then slightly lower again for the 30 wt% sample. A small amount of formate around 3% was consistently produced, which is not attributed to the gold catalyst as none was observed in the conventional H‐cell testing. With a reliable performance in triplicate experiments, the results show that the PVA loading can effectively tune the CO:H_2_ ratio. The maximum total yields based on the available Cr(II) charge is difficult to absolutely determine from state‐of‐charge studies and the consistent maximum yield of ca. 85% suggests a systematic loss of charge in the heterogeneous reaction. This was also observed in our previous work over bismuth nanomaterials to produce formate and hydrogen [6].

Figure S11 shows a TEM image of the 15 wt% PVA catalyst after a single pass of mediator. The gold nanoparticles have begun to agglomerate, although they are still individually defined. Because the particles are collected in a syringe filter after use, from which only a small amount can be recovered by sonication of the membrane, it is uncertain whether the agglomeration is a consequence of reaction degradation or simply an artefact of post‐mortem handling and analysis. The nature of the materials also made it challenging to assess the catalysts in a second pass, due to the low recovery when separating the material.

While we have not reused the mediator for repeated passes in this work, we are confident that it is not compromised by the reaction, with the given caveat that at very high pH the chromium will replace its PDTA ligand with OH^−^ and form a Cr(OH)_3_ colloid. This is however much outside the buffer region of the KHCO_3_ electrolyte, which is in excess compared to the mediator, so should not be a concern. The Cr PDTA complex, as well as some analogues, have been studied extensively by Marshak et al. Here, they report excellent stability and cyclability as a redox flow battery negolyte, with charge efficiency above 99.5% and no self‐discharge [21, 22]. This is however in borax buffer at a slightly higher pH of 9, which helps to further supress any competing HER during charging. The primary reason for avoiding reuse of the mediator herein was to mitigate the risk of catalyst cross‐contamination between experiments, thus ensuring that any changes observed in selectivity between catalysts were intrinsic. Regardless, UV–vis of the mediator solution post discharge appeared to show no apparent concentration change of the Cr PDTA complex, indicating high recyclability after initial use.

As an alternative method of catalyst preparation, a further series of materials were prepared in which the PVA was added after the impregnation of the gold nanoparticles onto the carbon. These materials were all prepared from the same batch of Au/C. The addition of PVA by this method had a similar effect for the first two PVA loadings tested as the material where the nanoparticles were formed in the presence of PVA. Increasing the loading, however resulted in no further improvement in CO selectivity, where all the loadings gave approximately the same 1:1 H_2_:CO ratio within error. A full table of results is given in the SI, Table S2.

A further set of samples were prepared from a commercial Au/C catalyst material by the same method, with the same 20% gold loading as the materials prepared in house. The addition of PVA to this material resulted in no improvement of CO selectivity within error, however. Consequently, these control samples unequivocally prove that the presence of PVA during nanoparticle formation is essential in creating a more selective CO_2_ reduction catalyst.

### 2.4 Towards Continuous Conversion and Online Analysis

In the initial screening method, only a small amount of the total CO_2_ was consumed during reduction, even if 100% FY was observed. Assuming a typical flask volume of 170 mL, the headspace volume after mediator injection would be 150 mL. Then, assuming a molar volume of 24.0 L mol^−1^ at 20°C and atmospheric pressure, this would result in 6.25 mmol of CO_2_ gas in the headspace. The solubility of CO_2_ in pure water is approximately 37 mmol/L under one atmosphere of pressure, adding a further 0.74 mmol of CO_2_, for approximately 7 mmol of total CO_2_ available. 20 mL of 10 mM Cr(II) mediator can provide 0.2 mmol of electrons, and assuming only two‐electron reduction products are observed, would allow for the conversion of 0.1 mmol of CO_2_, or 1.43% of the total CO_2_ available. Considering the FY observed for CO in the range of 40%–50%, the carbon conversion efficiency would be 0.57%–0.72%. Scaling the mediator concentration up to 0.1 M could result in the total possible CO_2_ conversion being 14.3%. Quantification of products for this level of conversion would present a considerable challenge due to saturation of the chromatography column and detector.

Because there is no counterbalancing half reaction in the batch system, the pH will be increased relative to the total number of electrons transferred. Whether by the intended catalysis or parasitic side reaction such as reduction of trace oxygen, the result of most of the possible reactions will include the formation of hydroxide. These will alter the pH of the electrolyte over time; however, this will be balanced to a degree by reaction with dissolved CO_2_ in the form of carbonic acid, resulting in the further consumption of the available CO_2_ to form carbonate or bicarbonate. Indeed, since the counter balancing Cr(II) to Cr(III) process releases no protons, each mole of two‐electron product H_2_ or CO will result in the consumption of one additional mole of CO_2_ lost to carbonate formation, limiting CO_2_ efficiency to 50% at best.

**Table:** 

Half reaction	*E* ^0^ versus SHE
$\mathrm{Cr}(\mathrm{III})\;\mathrm{PDTA}+\;e^{-}\to \;\mathrm{Cr}\;(\mathrm{II})\;\mathrm{PDTA}$	−1.1 V
$2H^{+}+2e^{-}\to \;H_{2}$	0 V
$2H_{2}O+\;2e^{-}\;\to \;H_{2}+\;2{\mathrm{OH}}^{-}$	−0.829 V
$2{{\mathrm{HCO}}_{3}}^{-}+2e^{-}\;\to \;H_{2}+\;2{{\mathrm{CO}}_{3}}^{2-}$	
${\mathrm{CO}}_{2}+2H^{+}+2e^{-}\to \;\mathrm{CO}+H_{2}O$	−0.1 V
${\mathrm{CO}}_{2}+2H_{2}O+\;2e^{-}\to \;\mathrm{CO}+\;2{\mathrm{OH}}^{-}$	−0.929 V
${\mathrm{CO}}_{2}+\;2{{\mathrm{HCO}}_{3}}^{-}+2e^{-}\to \;\mathrm{CO}+H_{2}O+2{{\mathrm{CO}}_{3}}^{2-}$	
$O_{2}+4H^{+}+\;4e^{-}\to \;2H_{2}O$	1.23 V
$O_{2}+2H_{2}O+4e^{-}\to \;4{\mathrm{OH}}^{-}$	0.401 V
**Reaction or equilibria**	**Relevant *K* value**
${\mathrm{CO}}_{2}+H_{2}O⇌H_{2}{\mathrm{CO}}_{3}$	*K*_h_ 0.0017
$H_{2}{\mathrm{CO}}_{3}+H_{2}O⇌H{{\mathrm{CO}}_{3}}^{-}+H_{3}O^{+}$	pKa 6.35
$H{{\mathrm{CO}}_{3}}^{-}+H_{2}O⇌{{\mathrm{CO}}_{3}}^{2-}+H_{3}O^{+}$	pKa 10.33
$H_{2}{\mathrm{CO}}_{3}+{\mathrm{OH}}^{-}\to H{{\mathrm{CO}}_{3}}^{-}+H_{2}O$	
$H{{\mathrm{CO}}_{3}}^{-}+{\mathrm{OH}}^{-}\to {{\mathrm{CO}}_{3}}^{2-}+H_{2}O$	

In an effort to determine if the DECO_2_R reaction can be practically scaled up, the concentration of the mediator was increased. At this concentration, CO_2_ mass transport and its overall availability is now likely to limit the reaction. To alleviate this, the system was modified with a continuous flow of CO_2_ through the electrolyte. This has the added benefit of allowing for semi‐continuous measurement of product concentration in the outflowing gas. The concentration of CO and H_2_ in the outflowing gas was measured every 15 min until the mediator was fully depleted. Completion was determined by a combination of colour change and the return of the O_2_ peak from trace air contamination introduced into the flow cell that could not be eliminated. While the mediator still processes charge, oxygen will be reduced, lowering the observed peak area in the GC trace. This will reduce the overall FY of the observed products.

Online batch DECO_2_R was performed using a round bottom flask as a continuous flow reactor. In the first instance, 20 mL of 0.1 M Cr PDTA mediator was injected into the reactor, which contained 2 mg of the Au/C 15% wt% PVA catalyst found to give the best CO selectivity of the materials tested. This would allow for a theoretical total of 1 mmol of two‐electron products, which if gaseous would occupy a volume of 24 mL at room temperature and pressure. A continuous flow of CO_2_ was passed through the reactor at a rate of 10 mL min^−1^, after which its composition was analysed by GC every 20 min.

**FIGURE 3:**
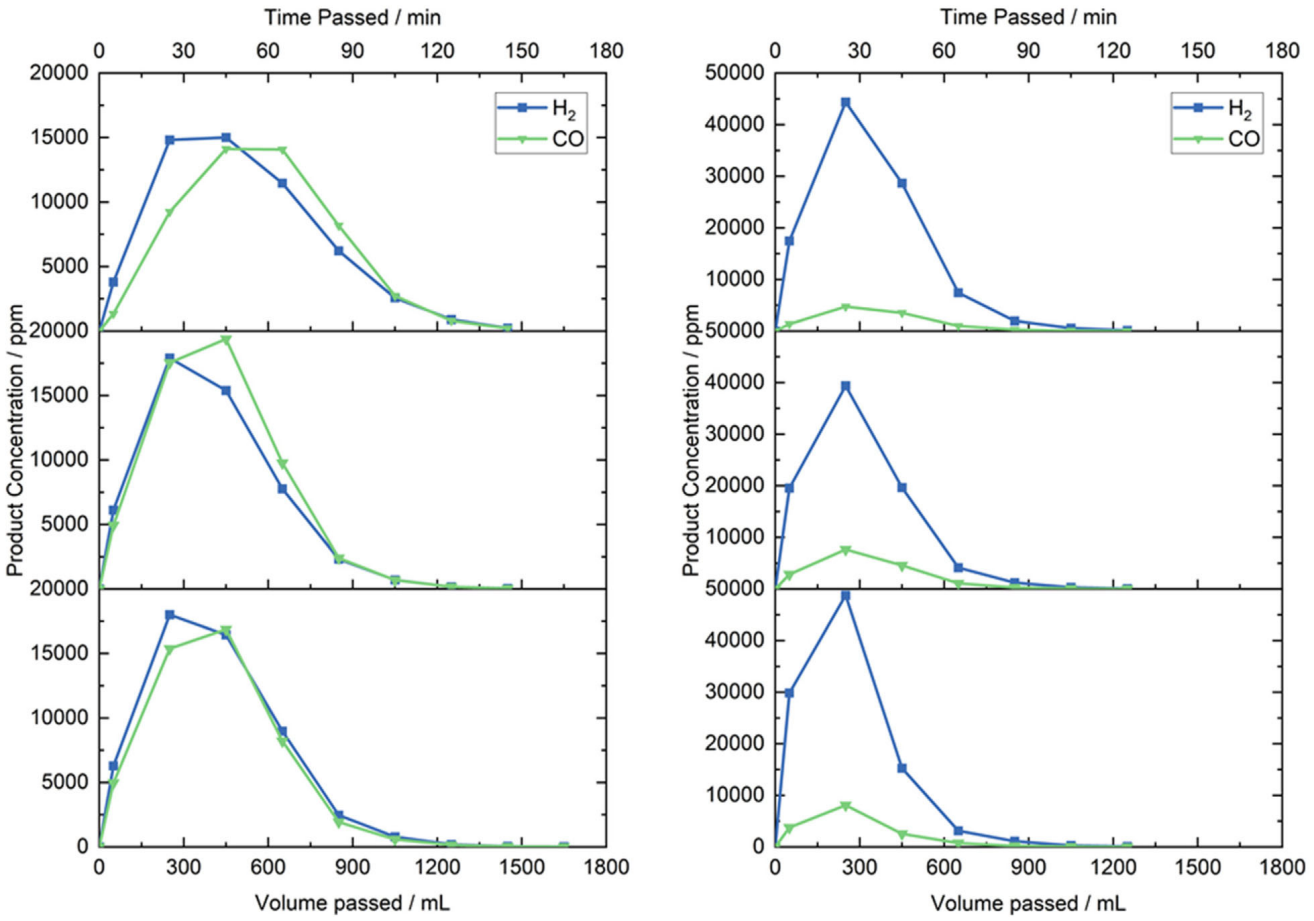
Evolution of the composition of the outflowing gas as a function of time and volume passed when 20 mL of 0.1 M Cr PDTA mediator was discharged over 2 mg of Au/C 15% wt% PVA catalyst under a continuous CO_2_ flow of 10 mL min^−1^. (Left) catalyst added as powder. (Right) catalyst added as dispersion in water. Each graph (left and right) shows the three repeats of the respective conditions.

Figure 3 (Left) shows how the composition of the outflowing gas changed over the course of the experiment. A rapid increase in product concentration is initially observed, which typically plateaus around the 40–60 min timestamp. The product formation then drops off quickly, with the mediator appearing fully depleted after about 100 −120 min, confirmed by the reappearance of trace O_2_ in the gas flow. The result was a syngas of roughly 1:1 H_2_ to CO, with FY of 42.4 ± 2.3%, 43.0 ± 1.2%, and 3.5 ± 0.1% for H_2_, CO and HCOO(H) respectively, for a total conversion of 88.8 ± 1.1%. Assuming the reaction was complete in 120 min, then the ‘current’ of the experiment was 26.8 mA, or the ‘current density’ was 13.4 mA mg^−1^. In this instance, current is approximated as the rate at which the capacity of the mediator is depleted. When multiplied by the observed FY, this would make the CO product specific current density 11.9 mA mg^−1^.

Conversely, when the same experiment was performed with the catalyst instead pre‐dispersed in 1 mL of water by sonication, the selectivity towards CO falls considerably, now producing a gas mixture with around 7:1 H_2_ to CO (Figure 3, Right). Overall FY was similar within error, with yields of 71.7 ± 3.7%, 12.9 ± 3.2%, and 3.3 ± 0.4% for H_2_, CO, and HCOO(H) respectively, for a total of 84.6 ± 4.8%. The rate was also much faster, reaching the plateau after just 30 min and with discharge effectively complete after just 60 min, doubling the apparent current.

**FIGURE 4:**
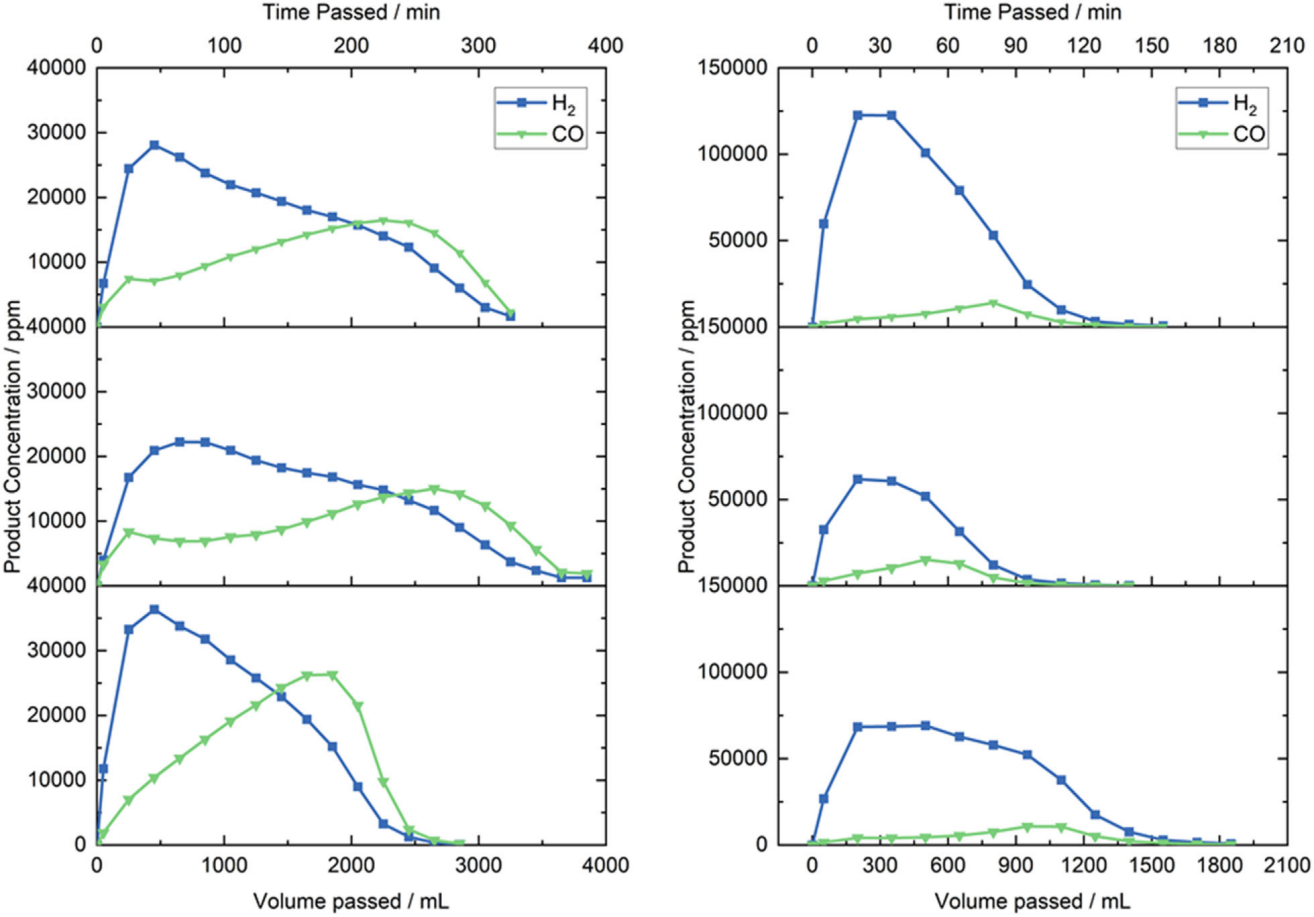
Evolution of the composition of the outflowing gas as a function of time and volume passed when 20 mL of 0.5 M Cr PDTA mediator was discharged over 2 mg of Au/C 15% wt% PVA catalyst under a continuous CO_2_ flow of 10 mL min^−1^. (Left) catalyst added as powder. (Right) catalyst added as dispersion in water. Each graph (left and right) shows the three repeats of the respective conditions.

The experiment was then scaled up even further, now using 20 mL of 0.5 M Cr PDTA mediator for a total possible 5 mmol of two‐electron products, or gaseous volume of 120 mL, with the results shown in Figure 4. A large initial spike in H_2_ production was observed, peaking around the 45 min mark and gradually falling as the discharge progressed. CO production on the other hand increased steadily as the discharge proceeded, only peaking as the mediator was almost discharged. The discharge was now prolonged to around 4–5 h, over the course of which an average of 52.7 ± 1.0 and 37.4 ± 2.0 mL of H_2_ and CO were produced respectively, for FY of 43.9 ± 0.9 and 31.1 ± 1.7%. HCOO(H) contributed a further 2.5 ± 0.2% for an overall FY of 77.6 ± 2.4%, which is somewhat lower than that observed for the 0.1 M case. The relative selectivity of H_2_ to CO was approximately 1.4:1, producing a syngas with a higher proportion of H_2_ than when 0.1 M mediator was used, but still comfortably within the industrially relevant range. A discharge time of 4–5 h relates to a current of 53.6–67.0 mA, or a current density 26.8–33.5 mA mg^−1^, around twofold that observed with 0.1 M mediator.

With the catalyst pre‐dispersed in water however, the discharge now took an average of 120 min, with the average current density now 67.0 mA mg^−1^. The amount of CO produced was consistent, at around 8.4 ± 0.1 mL, and out of a total possible volume of 120 mL, an average FY of 7%. The amount of H_2_ produced was on the other hand much less consistent, at 63.4 ± 19.6 mL, for an average FY of 53%. While the overall profile of product formation was largely similar between experiments, the maximum production rate varied considerably due to the difference in H_2_ produced. The amount of formate produced was consistent, accounting for about 1.2% of the FY across the set.

To understand the changes in both rate and selectivity observed when changing mediator concentration, we propose the possible rate equations as (1) and (2) for CO and H_2_ respectively. Depending on what is limiting the reaction rate, or ‘current’ passed, changing the mediator concentration could have different effects. Empirically the reaction rate corresponds to Equations (1) and (2).

(1) $$\frac{d\left[\mathrm{CO}\right]}{\mathrm{dt}}=k{\left[\mathrm{Cr}\left(\mathrm{II}\right)\right]}^{l}{\left[{\mathrm{CO}}_{2}\left(\mathrm{aq}\right)\right]}^{m}{\left[H^{+}\left(\mathrm{aq}\right)\right]}^{n}{\left[\mathrm{cat}\right]}^{p}$$

(2) $$\frac{d\left[H_{2}\right]}{\mathrm{dt}}=k{\left[\mathrm{Cr}\left(\mathrm{II}\right)\right]}^{l}{\left[H^{+}\left(\mathrm{aq}\right)\right]}^{n}{\left[\mathrm{cat}\right]}^{p}$$

If catalyst active sites, [cat], are the limiting factor, then production *rate* will not increase with mediator concentration, however if charge delivery is the limiting factor, then it will increase. It follows that when catalyst is limiting then product *selectivity* will not be dependent on available charge and should be unaffected by mediator concentration. However, if mediator charge delivery was limiting, then increasing mediator concentration will alter the reaction dynamics in such a way that changes the relative rates of the competing reactions. The only practical limits to the HER (Equation (2)) are active sites and rate of charge delivery because water is the solvent. However, the CO_2_R is also limited by the availability of CO_2_ at the catalyst surface (Equation (2)), and apparently, the nature of the catalyst dispersion.

As a considerable difference in selectivity, favouring HER, was observed when increasing [Cr(II)] from 0.1 to 0.5 M, it is apparent that the decoupled system is limited by the transport of CO_2_. The high selectivity observed in conventional H‐cell electrolysis could be due in part to the very low number of active sites and thus low current, even when using nanoparticle catalysts. It may also be the nature of the binding material and hydrophobicity of the catalyst when formed as an electrode. They are arranged on a 2D surface and usually require a Nafion or PTFE binder, meaning only a small number of the active sites will be exposed to the electrolyte. In a conventional system, charge delivery is effectively not limited (small effects from resistance are expected but can be compensated). In the decoupled system, it is likely the amount of exposed active sites is a magnitude higher, so with active sites no longer the limit, the product ratio is controlled by the transport of charge via the mediator and CO_2_ availability.

**FIGURE 5:**
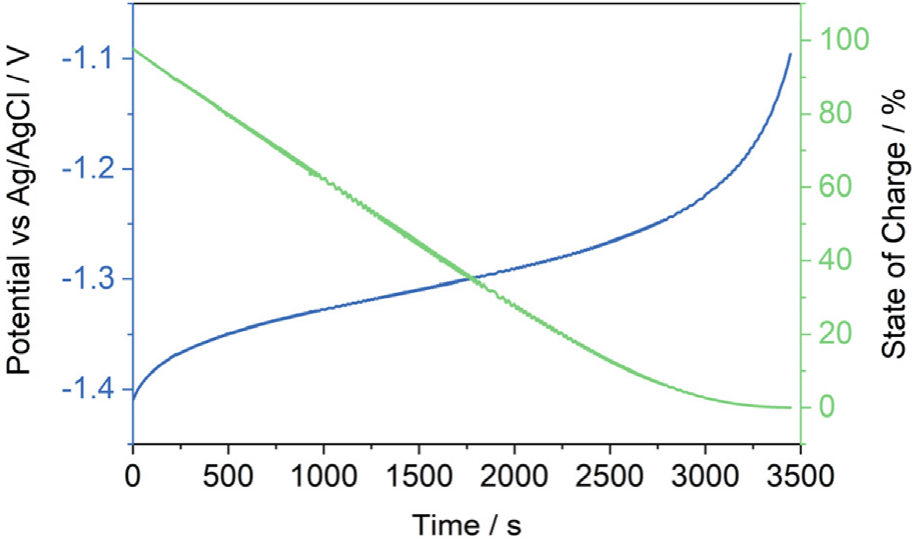
Example data of the continuous measurement of OCP during DECO_2_R by Au/C 15% wt% PVA with 0.1 M Cr PDTA mediator. The blue ‘Nernstian’ curve indicates the observed potential of the solution as a function of time, while the green curve indicates the corresponding state of charge calculated from the Nernst equation.

An initial exploration into the electrochemical kinetics of the reaction was performed using open circuit potential (OCP) as a measurement of state of charge (SOC) to uncover the rate at which electrons are transferred from the mediator to the catalyst (Figure 5). Electron transfer is governed by the concentration of reduced mediator, [Cr(II)], while the driving force of the reaction is governed by the Nernst Equation. However, the voltage profile during mediator discharge should be irrespective of initial concentration, as it should be controlled by the ratio of Cr(II)/Cr(III) rather than the total amount. The potential of the solution was measured using a glassy carbon working electrode compared to an Ag/AgCl reference electrode. To calibrate the response, the potential of a series of Cr(II)/Cr(III) mixtures was measured, which was found to give the expected Nernstian profile of potential as a function of Cr(II)/Cr(III) ratio. Despite this, a slight offset was noted in the *E* ^0^ measured from an equal mixture of Cr(II)/Cr(III) compared to the value obtained from cyclic voltammetry (CV) of the same solution, which unlike CV varied with mediator concentration, approaching the value from CV as concentration was increased.

A 10 cm^3^ aliquot of charged, CO_2_ saturated 0.1 mol dm^−3^ Cr PDTA was added to a small cell containing 1 mg of Au/C 15% wt% PVA catalyst. The OCP was measured continuously until the mediator was depleted, starting at ca −1.4 V and increasing to −1.1 V (Figure 5). The reaction proceeded at a constant rate for the first 90%, only slowing as the mediator became almost depleted. This would suggest that for the majority of the reaction, the rate is zeroth order with respect to the concentration of mediator, and the potential applied by the mediator (as controlled by the ratio of Cr(II)/Cr(III)). Under these conditions, either CO_2_ mass transport or active site availability dominate the observed rate. A crude estimate of rate can be calculated from the linear portion of the SOC versus time graph, where on average 68.8 ± 2.5% of the mediator was discharged after 2000s. This corresponds to a ‘volumetric current density’ of 3.32 ± 1.2 mA cm^−3^ mg^−1^, an absolute ‘current’ of 33.2 mA mg^−1^ catalyst or a production rate of 4.13 ± 0.15 μL s^−1^ assuming exclusively gaseous products.

We note that this is a condition‐specific rate, however, as CO_2_ concentration has been practically maximised, it is representative for the reaction under real conditions. From the online experiments we note that a small change in selectivity in favour of H_2_ was observed when mediator concentration was increased. As CO_2_R is contingent on both active sites and CO_2_ transport and HER only on active sites, it is likely that increasing mediator concentration has a greater effect in increasing the rate of HER than CO_2_R.

The near constant rate as determined by OCP measurement would appear to conflict with the varying production rate observed from the online GC experiments. This is attributed to the latency in GC analysis and the low temporal resolution of only three GC separations being completed within the reaction timeframe indicated from OCP measurement. Additionally, the requirement for manual dilution of samples into the GC calibration range further complicates the correlation of these methodologies. Both measurement techniques have their strengths, with the OCP measurement being immediate and continuous but offering no indication of the product(s) of the reaction. We anticipate that combining both techniques into a single experiment will allow for the collection of complementary data crucial to a deeper understanding of the kinetics of DECO_2_R.

### 2.5 Comparison to Conventional Systems

A brief reproduction of the conventional H‐cell electrolysis with continuous CO_2_ flow for the same catalyst material was performed for comparison with previously reported performance [13]. Controlled potential bulk electrolysis was performed at −0.7 V versus RHE (the approximate operating potential of the redox mediator) for a duration of 30 min. A summary of the results is given in Table S3, in which the selectivity for CO averaged 93% across the triplicate set, however the current density was merely 4.9 mA cm^−2^ (4.9 mA mg^−1^) while using a simple glassy carbon plate as the cathode. This was much lower than the previously reported value of 98.6 mA cm^−2^ (437 mA mg^−1^), which notably used a high surface area carbon/PTFE coated carbon paper microporous layer [13]. Given the disparity in performance the catalysts were tested within a conventional GDE system (Figures S14−16) using carbonate buffer. For electrolysis performed at −1.1 V versus SHE the current ranged between 12 and 27 mA cm^−2^ for a 10 cm^2^ cell with a catalyst loading of 1.2 mg cm^−2^ (10–23 mA mg^−1^). Such low current density was not unexpected at this potential, and while it compared favourably with the rate achieved by the decoupled system it was much lower than anticipated based on Ma et al.'s work. Furthermore, there appeared to be considerable degradation, not only in the first experiment where the current fell from 27 to 12 mA cm^−2^, but in the repeat experiment with the same electrode where current density steadily fell to just 6 mA after just 30 min of operation. Product yield and selectivity was initially high at 88% CO and 7% H_2_, however even over the first experiment, CO efficiency began to fall off rapidly, dropping to 69% at 30 min and 53% at 50 min. The corresponding H_2_ yield did not increase, resulting in a poor final Faradaic yield of just 57%. The repeat experiment saw a complete inversion of product selectivity alongside the decreased current density, with H_2_ now contributing the major product. Such a result highlights the non‐triviality of testing new catalyst materials even in existing systems, and that while GDEs offer a promising approach they are still limited heavily by stability issues.

As catalyst density was 1 mg cm^−2^ in the conventional testing, the current per milligram (mA/mg) used in the decoupled system is considered more comparable than current density (mA cm^−2^) traditionally reported. The ‘current’ observed with respect to catalyst mass in the decoupled system (33.2 ± 1.2 mA mg^−1^) was an order of magnitude higher than observed when we used the same material in a conventional H‐cell on a glassy carbon substrate, however this came at the cost of selectivity.

Industrially relevant GDE systems typically perform at current densities over 200 mA cm^−2^ [17, 23], but this is normally at high cell voltages. Such an approach cannot be replicated in the decoupled system, where applied potential is effectively governed by the SOC of the mediator. The Nernst equation dictates that a narrow range of potentials are practically attainable either side of the mediator formal potential. When GDE cells are operated comparatively at these lower potentials, much lower current densities below 100 mA cm^−2^ are typically observed, which do compare favourably with the decoupled system herein [24, 25, 26]. These references are pertinent as they specifically report the use of both HCO_3_^−^ supporting electrolyte and operation at potentials of −0.7 V versus RHE for the production of CO, thus providing a fair comparison to the decoupled system herein. We primarily highlight the works by Sargent et al., who used an analogous silver catalyst (rather than gold), and reported relatively poor current density in 1 M KHCO_3_ electrolyte of around 10 mA cm^−2^. This was considerably improved however upon switching to 1 M KOH [24] which is a consequence of enhanced cation promoter effects and CO desorption kinetics [27]. Indeed the current density of the silver catalyst is known to significantly vary as a function of electrolyte composition and operational parameters, as elucidated by Bhargava et al. [27]

We envision the use of a redox mediator to add another layer of tunability to the reaction conditions. In a conventional electrolyser, current density and applied potential are intrinsically linked, whereas in the decoupled system, current can be influenced by changing mediator concentration without changing the effective applied potential. In this way, conversion rates can be effectively increased without increasing the applied voltage, which typically results in increased degradation in conventional GDE systems. Of course, the true current limit of the decoupled system is dependent on the associated energy efficiency of the mediator charging step.

The pros and cons of the decoupled process are summarised in Table 1. We foresee the primary advantage of using a redox mediator is the inherent displacement of the catalyst from the electrode reactions and associated polarised interfaces. Indeed, a major energy efficiency limitation of conventional electrolysers at high current density are the ohmic losses caused by membrane, electrolyte and electrode assembly resistances [28]. This effect can be further exacerbated in the construction of a cell stack whereby ohmic losses are compounded. In the decoupled system, the charge is now delivered within the electrolyte alongside the other reactants, and as such the catalyst does not need to be electrically continuous and is no longer intrinsically limited to an essentially 2D interface. Because the mediator is able to both store and transfer its charge, the technology can be directly interfaced with energy storage through redox flow batteries to create a flexible system capable of power storage and chemical production concurrently. We accept that the ‘current’ delivered by the system will still be limited by the electrochemical charging step, however this can be optimised separately to take full advantage of state‐of‐the‐art flow battery architecture without having to consider the transport of multiple reactants or the collection of products within the electrochemical cell. Additionally, the activation overpotentials in flow batteries are typically smaller than those in CO_2_R GDE cells, when using kinetically facile redox mediators, which likely offers an enhancement of energy efficiency via the DECO_2_R approach.

**TABLE 1:** Summary of the pros and cons of decoupled electrolysis compared to conventional systems.

Advantage	Neutral	Disadvantage
No ohmic drop from electrode bound catalyst layer	Ion exchange membrane still needed in the mediator charging cell	Immature technology, may have as yet unknown limitations
Reactor stack would not have to be electrically connected	Still limited primarily by poor CO_2_ solubility	Small potential operating window defined by mediator
Allows for optimisation of a wholly aqueous electrochemical cell separately	Would still require coupled oxidation, most likely oxygen evolution	Catalyst immobilisation is required to prevent its ingress into the electrochemical cell and maintain decoupling
Integrates with electrical energy storage through dual function mediator		
Temperature and pressure of the cell and reactor can be controlled separately		

The DECO_2_R system will not fully avoid all limitations of conventional electrolyser design, however, as an electrolyte‐separating membrane will still be required in the mediator charging cell, with unavoidable associated ohmic losses. Further, CO_2_ transport is still a major hurdle to aqueous phase CO_2_R, primarily due to its low solubility, nor does it avoid the need for a counter process to replenish the oxidised mediator.

## 3 Conclusions and Future Work

The emerging paradigm of decoupled electrochemical reduction has been demonstrated for the first time in the production of syngas from CO_2_, using a reducing Cr(II) PDTA mediator and a PVA‐capped gold catalyst. Cr(II) species were generated in a conventional electrochemical flow cell, while CO_2_R was executed in a separate vessel utilising the potential of the charged electrolyte. The work successfully extends the concept of DECO_2_R to the production of gaseous products, CO and H_2_, which are easily separated from the discharged mediator solution. The use of PVA as a capping agent for the gold nanoparticles proved highly effective in boosting selectivity towards CO and enabling efficient conversion at the modest reduction potential of ca. −0.7 V versus RHE provided by the Cr PDTA mediator. By varying the amount of PVA, a range of CO/H_2_ ratios were obtained spanning (2:1–1:1), with the highest FY observed for DECO_2_R approaching 90%.

An initial exploration into scaling up the decoupled process by increasing the mediator concentration, indicated that current densities approaching state of the art systems at low potentials can be realised. Despite this, the mass transport of dissolved CO_2_ still appears to be the limiting factor, with higher mediator concentration resulting in a lower selectivity towards CO. Further, the method of catalyst addition (dry vs. wet) resulted in considerable change to the relative selectivity. The wet, pre‐dispersed addition produced far more hydrogen, suggesting that catalyst presentation is a crucial component defining selectivity. Nevertheless, the DECO_2_R approach for CO production, as an alternative to conventional electrolysis, is promising because of unique operational advantages that may prove more easily implemented at scale. Brief comparison to a GDE system operated at a similar overpotential indicated that while some selectivity towards CO is lost, similar current density can be achieved specifically in KHCO_3_ electrolyte. As a CO_2_R product, CO would primarily be considered as an intermediate in the production of a wide range of chemicals by reaction alongside H_2_ such as via the Fischer–Tropsch process. Herein the syngas H_2_/CO ratio was found to be directly controllable as a function of the catalyst composition and mediator state‐of‐charge, offering tailored production of syngas mixtures by system reconfiguration, as opposed to varying the cathode voltage in conventional electrolysers.

These preliminary results show there is promise to the alternative approach of decoupled electrolysis, providing temporal and spatial decoupling of complex reaction processes. To further develop the DECO_2_R concept into a practical system, a reactor framework that allows the catalyst to be used indefinitely under either continuous flow or sequential batch reactions must be developed. Optimisation of process design and reaction conditions will undoubtedly lead to better performances, as they have with conventional electrolysers.

## Author Contributions

**Mark Potter:** conceptualisation (supporting), data curation (lead), formal analysis (lead), investigation (lead), methodology (lead), project administration (supporting), validation (lead), visualisation (lead), writing – original draft (lead), writing – review and editing (lead). **Hamza Annath:** data curation (supporting), formal analysis (supporting), investigation (supporting), methodology (equal), writing – original draft (supporting). **Craig G. Armstrong:** data curation (supporting), resources (supporting), writing – review and editing (supporting). **Marina Goupalova:** data curation (supporting). **Enrico Andreoli:** funding acquisition (supporting), resources (supporting), writing – review and editing (supporting). **Kathryn E. Toghill:** conceptualisation (lead), funding acquisition (lead), project administration (lead), resources (lead), supervision (lead), writing – review and editing (equal).

## Funding

This work was supported by HORIZON2020 European Research Council (Starting Grant 805344 DeCO‐HVP) and Engineering and Physical Sciences Research Council (Grant EP/S018107/1).

## Conflicts of Interest

The authors declare no conflicts of interest.

## Supporting information

Supplementary material

## Data Availability

The data that support the findings of this study are openly available in [Zenodo] at [https://doi.org/10.5281/zenodo.17609833].
