# Supplementary material for: Rapid Decoupled Electrochemical Reduction of CO2 to Syngas
Source: ChemSusChem. 2026 Jul 11;19(14):e202502728. doi: 10.1002/cssc.202502728 (PMC13354969; doi:10.1002/cssc.202502728)
Supplement: Supplementary file 1 — Supplementary material [file CSSC-19-e202502728-s001.pdf]

# Rapid Decoupled Electrochemical Reduction of $CO_2$ to Syngas

Supplementary Information

Mark Potter, Hamza Annath, Craig G. Armstrong, Marina Goupalova, Enrico Andreoli and Kathryn E. Toghill*

[a] Department of Chemistry, Lancaster University, Lancaster, LA1 4YB

E-mail: k.toghill@lancaster.ac.uk

[b] Department of Chemical Engineering, Swansea University, Bay Campus, Swansea, SA1 8EN, UK

Formatted: Italian (Italy)

## Experimental

### Chemicals

All chemicals were used as purchased without further purification. Sodium borohydride (99%), carbon black (99.9%), sodium chloride (99.5%), potassium chloride (99.5%), potassium ferrocyanide (99%), potassium hydrogen carbonate (99.7%), and acetone (99%) were purchased from Thermo Fisher (Alfa Aesar, Acros Organics). Chloroauric acid trihydrate (99.9%), polyvinyl alcohol (9000-10000 mw, 80% hydrolysed), chromium potassium sulphate dodecahydrate, 1,3-propanediamine-N,N,N',N'-tetraacetic acid (PDTA, 99%), and potassium hydroxide (reagent grade) were purchased from Merck (Sigma Aldrich). $CO_2$ was purchased from BOC ltd. Isopropanol (99.5%) was purchased from Honeywell.

### Synthesis

KCrPDTA was synthesised following previously reported procedure.[1,2] In brief, $KCr(SO_4)_2 \cdot 12H_2O$ (29.6 g, 59.3 mmol) was dissolved in water (120 mL) alongside $H_4$-PDTA (18.4 g, 60.1 mmol), and the resulting purple solution was refluxed for 4 hours. The resulting red solution was partially neutralised by the addition of KOH flakes (12 g), and was refluxed for a further 16 hours overnight. The solution was then removed from the heat and fully neutralised by dropwise addition of 1 M KOH. To this, an equal volume of acetone (around 200 mL) was added, resulting in immediate formation of an off-white precipitate which was removed by vacuum filtration ($K_2SO_4$). The Volume of the filtrate was then reduced to around 60 mL by rotary evaporation, after which it was added slowly to ice cold isopropanol (350 mL), resulting in a dark red microcrystalline precipitate. This was collected by vacuum filtration and dried at 70°C. The isolated crystals are $KCrPDTA \cdot 3H_2O$, M.W. 447 g $mol^{-1}$.

Gold nanoparticles supported on carbon black (20 wt.% gold) with polyvinyl alcohol capping were synthesised by a straightforward two-step synthesis adapted from existing literature.[3] Gold nanoparticles were initially formed by the rapid reduction of $HAuCl_4$ solution (1 mM, 200 mL) by the addition of $NaBH_4$ (0.1 M, 10 mL) in the presence of PVA (added as a 1 wt.% solution), after which carbon black (160 mg) was added along with NaCl (1.2 g) to destabilise the dispersion and hasten the impregnation. The salts were then removed by centrifugation and redispersion in DI water, which was then evaporated at 50°C resulting in a fine black powder.

**Electrochemical methods**

Electrochemical experiments were performed using a Biologic SP300 potentiostat or Ivium Vertex 5A. Voltammetry was performed in a small glass cell of approximately 20 mL volume, using a 3 mm diameter glassy carbon working electrode unless otherwise stated. For aqueous experiments, the reference electrode was typically Ag/AgCl, using 3 M NaCl inner solution. The potentials of the reference electrode was periodically checked against a saturated calomel reference electrode to compensate for any potential drift over time, and all potentials reported against RHE throughout the work were calculated related to this.

To access the Cr(II) charged state of the mediator, a large electrochemical flow cell with geometric electrode areas of 16 $cm^2$ was used to charge the electrolytes for decoupled $CO_2$ reduction and to test performance of the electrolytes as a cell. The Cr(III) PDTA was charged against excess $Fe(II)(CN_6)$ as a sacrificial electron source. The mediator was overcharged to the point of hydrogen evolution in the cell to ensure ca. 100% charging for decoupled experiments. The charged electrolyte was thoroughly saturated with $CO_2$ (or $N_2$ for controls) before use to eliminate any residual $H_2$. The cell was constructed from an outer frame of steel, polypropylene electrolyte diffusers, brass current collectors, graphite composite electrode plates, graphite felt electrodes, EPDM gaskets, Fumatek Fumapem F-930 cation exchange membrane.

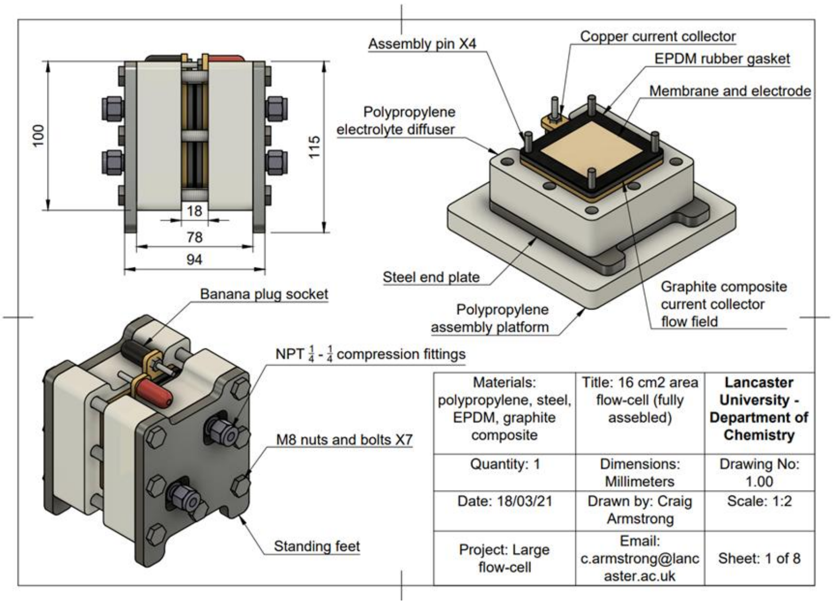


*Figure S1. Schematic representation of the 16 cm$^2$ geometric area flow cell used to access the charged state of the mediator.*

**Batch DE$CO_2$R**

The initial results were obtained from a batch reaction performed in a Schlenk flask. Air was removed from the flask, containing the catalyst as a powder, by alternating between vacuum (<1 mBar) and $CO_2$ (or $N_2$ in the case of controls) 5x to eliminate as much oxygen a reasonably possible. To this, mediator solutions saturated with $CO_2$ (or $N_2$) were injected through a PTFE faced silicone septum. The resulting $CO_2$R products were analysed by a combination of gas and ion chromatography.

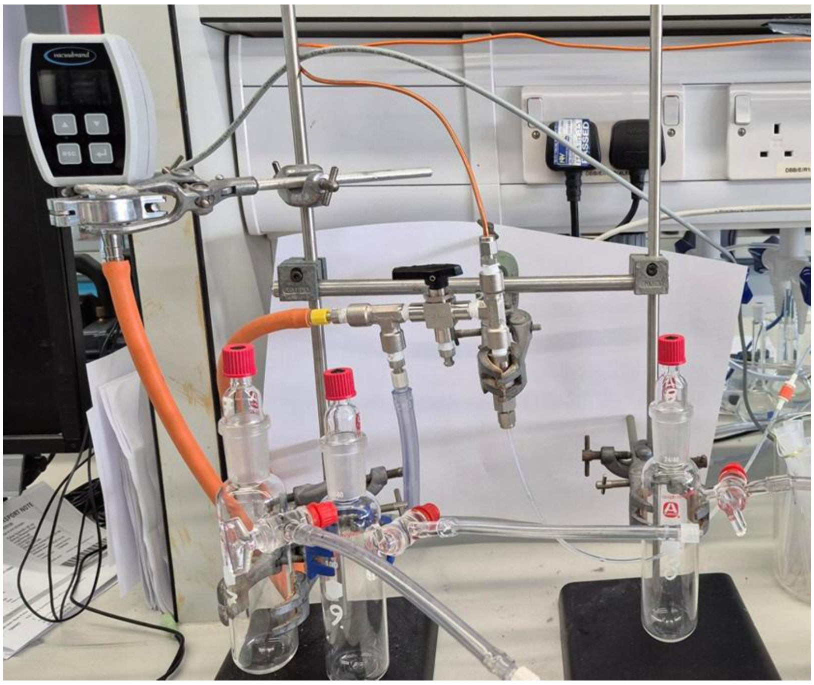

*Figure S2. Experimental setup used for the batch screening of the Au/C catalysts for DE$CO_2$R.*

**DE$CO_2$R with online GC**

As an initial design, a three necked round bottom flask was used as a flow reactor. In this instance, only the gas was flowing. $CO_2$ was injected through one arm of the flask by a needle submerged below the electrolyte surface. A second needle in the other arm allowed headspace gas to flow out of the flask and through the injection loop of the GC where its composition was analysed every 20 minutes. Mediator and catalyst were added by the same manner as described for the batch reactor design. This allowed for the collection of semi-quantitative rate data, as the composition of the outflowing gas reflected the rate of production. The volume of the flask was measured to be 163.15 $cm^3$.

**H-Cell Electrolysis**

H-cell electrolysis was performed in a custom glass H-cell. The volume of the working compartment was approximately 87 mL, of which 7 mL was taken up by electrodes, 40 mL by electrolyte, and 40 mL by gas headspace. The working compartment was separated from the counter by a Fumapem F-930 cation exchange membrane. The working electrode consisted of a glassy carbon plate with a confined area of 1.2x1.2 cm, for 1.44 $cm^2$, onto which the

catalyst was drop cast as an ink. The connecting copper wire is plastic coated to avoid contamination.

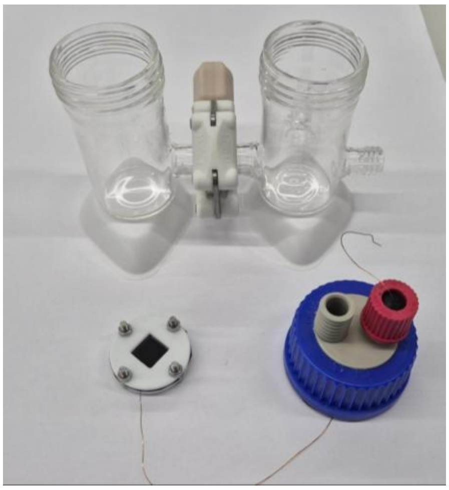

*Figure S3. H-cell components*

**GDE Electrolysis**

GDE experiments were conducted by Swansea University using their in-house designed 10 $cm^2$ cell.[4] Electrolysis was performed potentiostatically using a Ag/AgCl reference electrode, 1 M $KHCO_3$ electrolytes pumped at 100 mL $min^{-1}$ and a reactant $CO_2$ flow rate of 80 mL $min^{-1}$. The GDE was operated at a differential pressure of 0 ± 5 mbarD with $CO_2$ and electrolyte pressures of approximately 110 mbar. Gaseous products were analysed by online GC-TCD-FID and faradaic yields were calculated for the corresponding current density at time of sample injection. The GDE was prepared from a catalyst ink that was prepared by mixing: 6 g iPrOH, 6 g $H_2O$, 106 mg Nafion D520 (5 wt% solution), and 48 mg Au/C 15% PVA catalyst powder. The ink was sonicated for 20 min using an ultrasonic probe (500 W) at 20 % amplitude and a program of continuous 5 s power followed by 5 s cooling. Total time of 40 min and max temperature of approximately 60 °C. The ink was stirred during sonication. The ink was sprayed onto a Freudenberg H15C13 gas diffusion layer (40 x 40 $mm^2$) using a 0.3 mm manual airbrush and a hotplate at 140 °C. The catalyst layer loading was measured giving: 1.34 mg $cm^{-2}$ total loading, 1.2 mg $cm^{-2}$ catalyst loading (90 wt%), 0.13 mg $cm^{-2}$ Nafion loading (10 wt%).

**Product quantification**

Calibrations were prepared to allow for the quantification of the products $H_2$, CO, $CH_4$, and $C_2H_4$. The gases were analysed on a Shimadzu 2030 GC system equipped with a ResTek ShinCarbon ST 80/100 column and barrier ionisation discharge (BID) detector, using helium as the eluent gas. Linear calibrations in the range 200-1,000 ppm were prepared for all four gases, with further curved calibrations in the range 2,000-100,000 prepared for $H_2$ and CO. Certified calibration gas standards were purchased from BOC ltd. The range of concentrations were prepared by dilution of these standards with $CO_2$ by the use of two gas tight syringes.

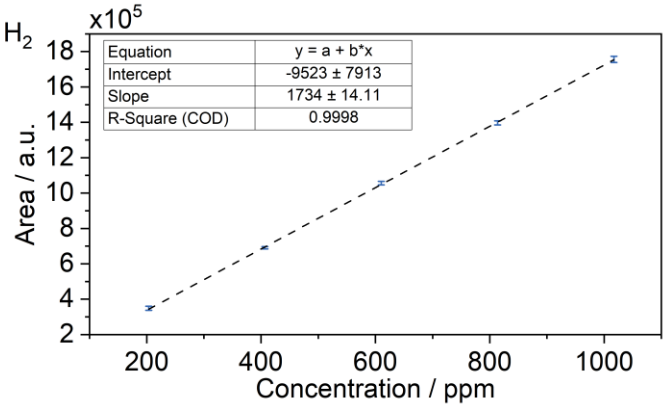


*Figure S4. Calibration plot for $H_2$ quantification in the range 200-1,000 ppm.*

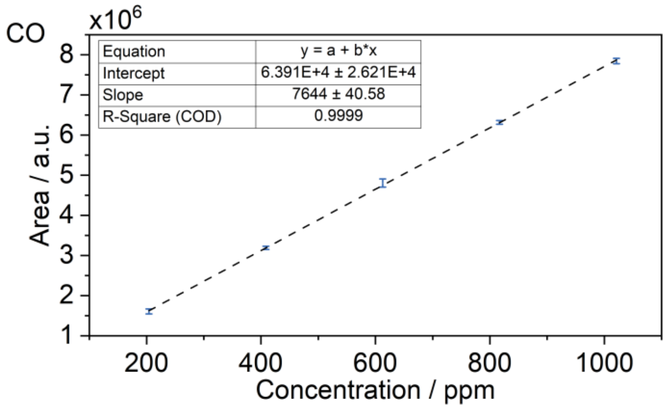


*Figure S5. Calibration plot for CO quantification in the range 200-1,000 ppm.*

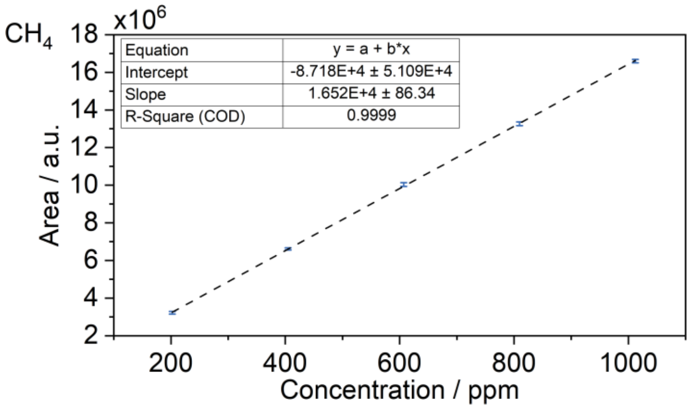


*Figure S6. Calibration plot for $CH_4$ quantification in the range 200-1,000 ppm.*

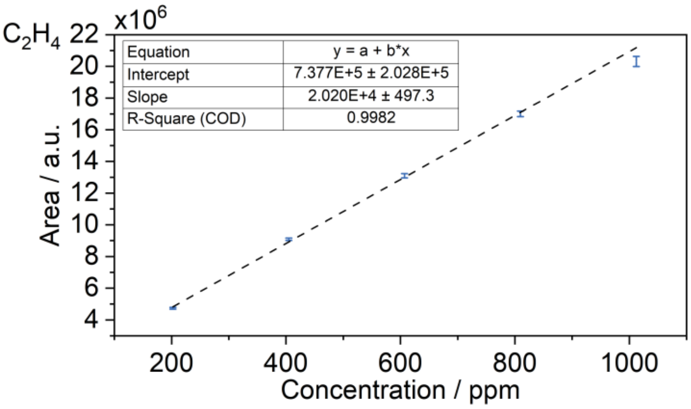


*Figure S7. Calibration plot for $C_2H_4$ quantification in the range 200-1,000 ppm.*

As an example, a 5-fold dilution was achieved by taking 10 mL of sample into the first syringe and 40 mL of $CO_2$ into the second, after which the gases were transferred between the syringes 5 times to ensure thorough mixing. The confidence of the linear calibration is high, with $R^2$ values >0.99 in the 200-1,000 ppm range for all gases.

The high ppm (2,000-100,000 ppm) calibrations were fitted to a curve approximated by a one phase association:

$$y = y_0 + (Plateau - y_0)(1 - e^{-kx^a})$$

Where *plateau* corresponds to the maximum y value, $y_0$ corresponds to the intercept, and the values of the constants *k* and *a* were optimised to minimise the error calculated from the sum

of the square of the difference between real and predicted *y* values for each concentration point.

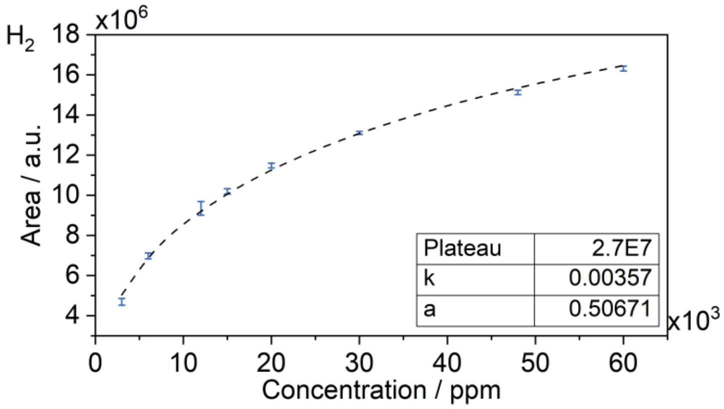


*Figure S8. Calibration plot for $H_2$ quantification in the range 3,000-60,000 ppm.*

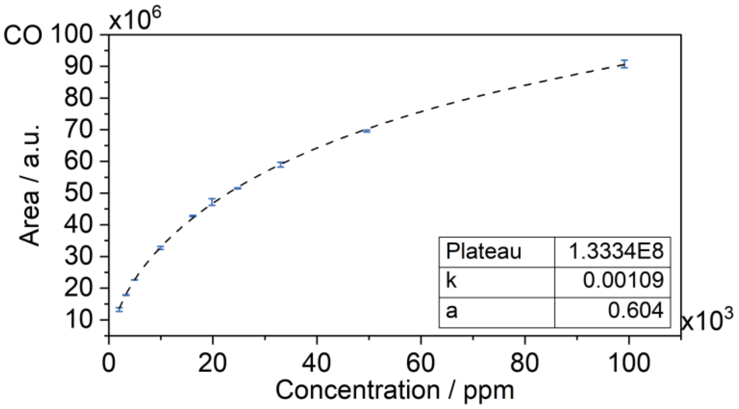


*Figure S9. Calibration plot for CO quantification in the range 2,000-100,000 ppm.*

With these calibrations, the concentration of the product gases in the headspace of the reaction vessel were measured. Knowing the total volume of each reactor and the volume of electrolyte within, the total amount of products formed can be estimated. It was assumed that the amount of each product dissolved in the electrolyte was negligible as they are all sparingly soluble.

From this, the faradaic efficiency for each product was determined.

The amount of each gaseous product in moles within the headspace was estimated;

$$\textit{Moles product [mol]} =$$

$$\frac{\textit{Headspace concentration [ppm]}}{10^6} \times \textit{Moles of gas in headspace [mol]}$$

where the ideal gas law was used to determine the total number of moles within the headspace;

$$\textit{Total moles of gas in headspace} =$$

$$\frac{\textit{Headspace pressure [Pa]} \times \textit{Headspace volume } [m^3]}{\textit{Ideal gas constant } [J\ K^{-1}\ mol^{-1}] \times \textit{Temperature [K]}}$$

By considering the electron stoichiometry, the amount of charge needed to form each product can be calculated;

$$\textit{Charge required [C]} =$$

$$\textit{Moles product [mol]} \times \textit{Electron stoichiometry} \times \textit{Faraday constant } [C\ mol^{-1}]$$

which can then be compared to the total charge passed;

$$\textit{Charge passed [C]} =$$

$$\textit{Moles of mediator [mol]} \times \textit{Electron stoichiometry} \times \textit{Faraday constant } [C\ mol^{-1}]$$

to determine the faradaic efficiency as a quantum yield;

$$\textit{Faradaic efficiency} = \frac{\textit{Charge required [C]}}{\textit{Charge passed [C]}} \times 100$$

The same approach was employed for the liquid phase product HCOO(H), with the product moles calculated assuming a sample density of 1 kg $L^{-1}$

$$\text{Moles product [mol]} =$$

$$\frac{\text{Measured Concentration [ppm]}}{\text{Molecular mass } [\text{Kg mol}^{-1}]} \times \text{Total volume of electrolyte [L]}$$

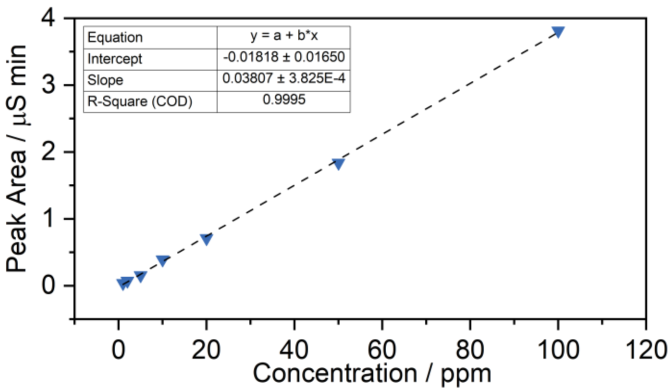


*Figure S10. Calibration plot for HCOO(H) quantification in the range 1-100 ppm.*

**Results**

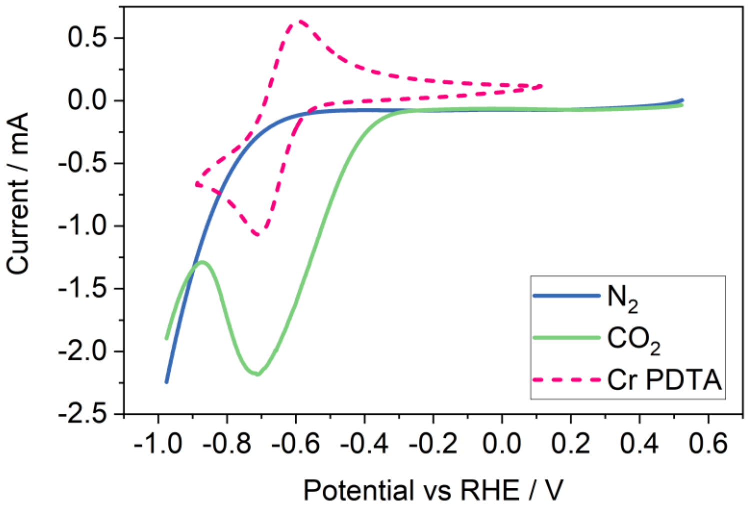


***Figure S11.*** *LSV of Au/C 15 wt.% PVA coated GC electrode in 1 M KCl. CV of 10 mM Cr PDTA overlayed to show potential overlap.*

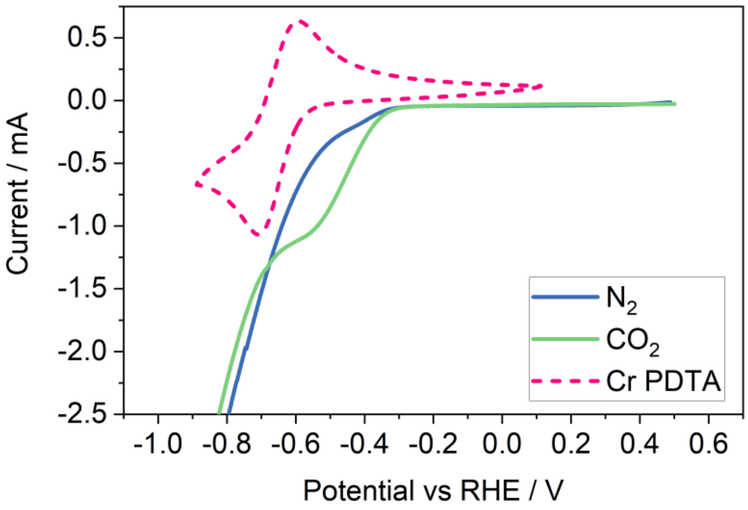


***Figure S12.*** *LSV of Au/C 15% wt.% PVA coated GC electrode in 1 M $KHCO_3$. CV of 10 mM Cr PDTA overlayed to show potential overlap.*

*Table S1. Results of conventional H-cell $ECO_2R$ using Au/C 7.5% wt.% PVA cast onto a glassy carbon plate working electrode of area 1.44 $cm^2$. Potentials are referenced vs RHE assuming a pH of 7.5 for $CO_2$ saturated 1 M $KHCO_3$. Drop casting was achieved using a Nafion binder with a catalyst to binder ratio of 9:1 and a density of 1 mg $cm^{-2}$.*

| **Potential (V) vs RHE** | **FY (H2) %** | **FY (CO) %** | **Total FY %** | **Relative CO %** | **Total Charge passed (C)** | **Average current density (mA $cm^{-2}$)** |
|---|---|---|---|---|---|---|
| -0.856 | 16.5 | 59.5 | 76.0 | 78 | 6.23 | 2.4 |
| -0.756 | 15.8 | 63.9 | 79.7 | 80 | 4.96 | 1.9 |
| -0.656 | 8.0 | 70.7 | 78.7 | 90 | 3.65 | 1.4 |
| -0.556 | 23.2 | 67.5 | 90.7 | 74 | 2.72 | 1.1 |

*Table S2. Faradaic yields and product selectivity observed using the residually capped Au/C PVA catalysts.*

| **Au/C Catalyst** | **Overall Faradaic Yeld (%)** | **Product ratio (% of observed products)** | | |
|---|---|---|---|---|
| | | $H_2$ | CO | $HCOO^-$ |
| **Commercial Au/C** | | | | |
| **no PVA** | 77.80 ± 0.8 | 75.95 ± 2.66 | 20.33 ± 2.72 | 3.72 ± 0.21 |
| **2.5% wt.% PVA** | 77.86 ± 2.0 | 74.88 ± 4.89 | 22.33 ± 4.75 | 2.79 ± 0.16 |
| **7.5% wt.% PVA** | 77.42 ± 1.1 | 76.18 ± 2.33 | 21.03 ± 2.34 | 2.79 ± 0.10 |
| **In-house Au/C** | | | | |
| **0% wt.% PVA** | 83.7 ± 2.3 | 75.8 ± 1.2 | 21.0 ± 1.0 | 3.3 ± 0.19 |
| **2.5% wt.% PVA** | 77.5 ± 1.7 | 52.46 ± 2.09 | 44.29 ± 2.36 | 3.25 ± 0.30 |
| **5.0% wt.% PVA** | 75.7 ± 0.21 | 44.02 ± 2.95 | 53.03 ± 2.95 | 2.95 ± 0.06 |
| **7.5% wt.% PVA** | 81.96 ± 0.53 | 46.22 ± 0.79 | 49.44 ± 0.90 | 4.34 ± 0.12 |
| **10.0% wt.% PVA** | 80.02 ± 1.5 | 47.42 ± 0.62 | 48.76 ± 0.75 | 3.82 ± 0.23 |
| **15.0% wt.% PVA** | 81.8 ± 01.1 | 47.47 ± 2.48 | 48.35 ± 2.62 | 4.18 ± 0.15 |

*Table S3. Results of conventional H-cell $ECO_2R$ using Au/C 15% PVA cast onto a glassy carbon plate working electrode of area 1.44 $cm^2$ with continuous $CO_2$ flow (10 $cm^3$ $min^{-1}$). Drop casting was achieved using a Nafion binder with a catalyst to binder ratio of 9:1 and a density of 1 mg $cm^{-2}$. A potential of −0.7 V vs RHE (assuming a pH of 7.5 for $CO_2$ saturated 1 M $KHCO_3$) was applied for 30 minutes. Faradaic yields of $H_2$ and CO were determined from post-analysis of the gas collected in a Tedlar® bag.*

| | 1 | 2 | 3 | Average |
|---|---|---|---|---|
| **Charge Passed (C)** | 11.0 | 14.0 | 12.7 | 12.6 |
| **$H_2$ FY (%)** | 6.0 | 3.7 | 6.9 | 5.5 |
| **CO FY (%)** | 79.5 | 77.2 | 83.0 | 79.9 |
| **Total FY (%)** | 85.5 | 81.0 | 89.9 | 85.5 |
| **Current Density (mA $cm^{-2}$)** | 4.3 | 5.4 | 4.9 | 4.87 |

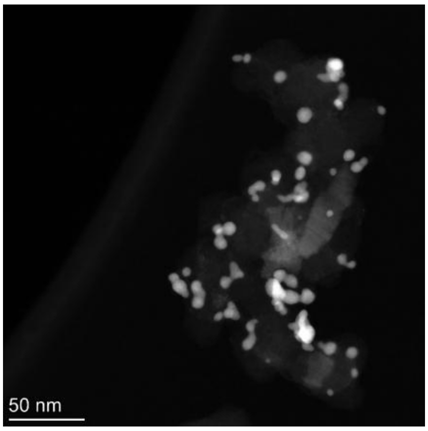


*Figure S13. Spent Au/C 15% wt.% PVA displaying some agglomeration of nanoparticles after being deployed for $DECO_2R$*

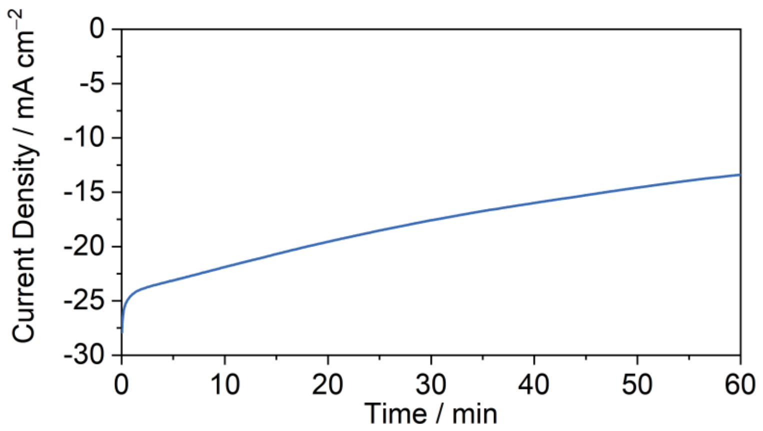


*Figure S14. Chronoamperometry of Au/C 15% wt.% PVA in a GDE cell configuration, displaying the current density as a function of time while held at a constant potential of −1.1 V vs SHE. The cell was operated against the oxidation of Ferrocyanide as sacrificial electron source, using 1 M $KHCO_3$ supporting electrolyte. Experiment run 1.*

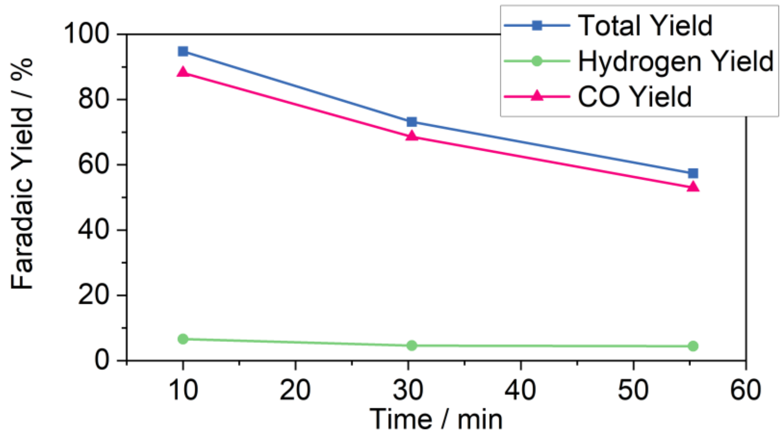


*Figure S 15. Measured Faradaic yield for the products CO and $H_2$ as a function of time for the electrolysis experiment shown in Figure S14.*

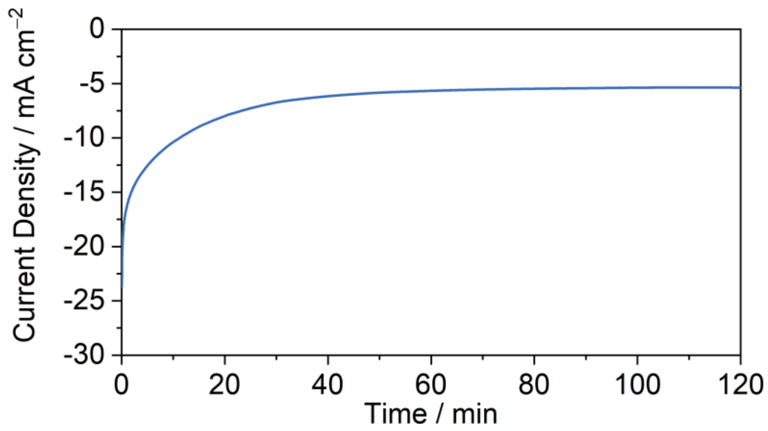


*Figure S16. Chronoamperometry of Au/C 15% wt.% PVA in a GDE cell configuration, displaying the current density as a function of time while held at a constant potential of −1.1 V vs SHE. The cell was operated against the oxidation of Ferrocyanide as sacrificial electron source, using 1 M $KHCO_3$ supporting electrolyte. Experiment run 2.*

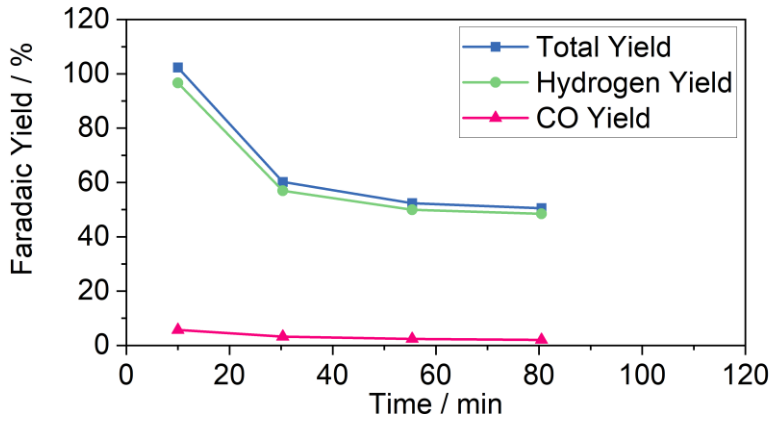


*Figure S17. Measured Faradaic yield for the products CO and $H_2$ as a function of time for the electrolysis experiment shown in Figure S16.*

**References**


[1] B. H. Robb, J. M. Farrell, M. P. Marshak, Chelated Chromium Electrolyte Enabling High-Voltage Aqueous Flow Batteries, *Joule* **2019**, *3*, 2503–2512.

[2] M. Potter, D. E. Smith, C. G. Armstrong, K. E. Toghill, Electrochemically decoupled reduction of CO2 to formate over a dispersed heterogeneous bismuth catalyst enabled via redox mediators , *EES Catal.* **2024**, *2*, 379–388.

[3] L. Ma, W. Hu, Q. Pan, L. Zou, Z. Zou, K. Wen, H. Yang, Polyvinyl alcohol-modified gold nanoparticles with record-high activity for electrochemical reduction of CO2 to CO, *J. CO2 Util.* **2019**, *34*, 108–114.

[4] C. G. Armstrong, B. Sandnes, E. Andreoli, Sub-millibar pressure gradient along a gravity-driven percolated CO2 gas diffusion electrode for vertical scale-up, *Ind. Chem. Mater.* **2026**, DOI 10.1039/D5IM00372E.
